# Minicircle DNA-mediated endothelial nitric oxide synthase gene transfer enhances angiogenic responses of bone marrow-derived mesenchymal stem cells

**DOI:** 10.1186/s13287-016-0307-2

**Published:** 2016-04-01

**Authors:** Nadeeka Bandara, Saliya Gurusinghe, Haiying Chen, Shuangfeng Chen, Le-xin Wang, Shiang Y. Lim, Padraig Strappe

**Affiliations:** School of Biomedical Sciences, Charles Sturt University, Wagga Wagga, NSW 2650 Australia; Central laboratory and key Laboratory of Oral and Maxillofacial-Head and Neck Medical Biology, Liaocheng People’s Hospital, Liaocheng, 252000 PR China; O’Brien Institute Department, St. Vincent’s Institute of Medical Research, Fitzroy, VIC 3065 Australia; Department of Surgery, St. Vincent’s Hospital, University of Melbourne, Melbourne, VIC 3002 Australia

**Keywords:** Minicircle, DNA vector, Transfection, Endothelial nitric oxide synthase, Mesenchymal stem cells, Nitric oxide, Angiogenesis

## Abstract

**Background:**

Non-viral-based gene modification of adult stem cells with endothelial nitric oxide synthase (eNOS) may enhance production of nitric oxide and promote angiogenesis. Nitric oxide (NO) derived from endothelial cells is a pleiotropic diffusible gas with positive effects on maintaining vascular tone and promoting wound healing and angiogenesis. Adult stem cells may enhance angiogenesis through expression of bioactive molecules, and their genetic modification to express eNOS may promote NO production and subsequent cellular responses.

**Methods:**

Rat bone marrow-derived mesenchymal stem cells (rBMSCs) were transfected with a minicircle DNA vector expressing either green fluorescent protein (GFP) or eNOS. Transfected cells were analysed for eNOS expression and NO production and for their ability to form in vitro capillary tubules and cell migration. Transcriptional activity of angiogenesis-associated genes, CD31, VEGF-A, PDGFRα, FGF2, and FGFR2, were analysed by quantitative polymerase chain reaction.

**Results:**

Minicircle vectors expressing GFP (MC-GFP) were used to transfect HEK293T cells and rBMSCs, and were compared to a larger parental vector (P-GFP). MC-GFP showed significantly higher transfection in HEK293T cells (55.51 ± 3.3 %) and in rBMSC (18.65 ± 1.05 %) compared to P-GFP in HEK293T cells (43.4 ± 4.9 %) and rBMSC (15.21 ± 0.22 %). MC-eNOS vectors showed higher transfection efficiency (21 ± 3 %) compared to P-eNOS (9 ± 1 %) and also generated higher NO levels. In vitro capillary tubule formation assays showed both MC-eNOS and P-eNOS gene-modified rBMSCs formed longer (14.66 ± 0.55 mm and 13.58 ± 0.68 mm, respectively) and a greater number of tubules (56.33 ± 3.51 and 51 ± 4, respectively) compared to controls, which was reduced with the NOS inhibitor L-NAME. In an in vitro wound healing assay, MC-eNOS transfected cells showed greater migration which was also reversed by L-NAME treatment. Finally, gene expression analysis in MC-eNOS transfected cells showed significant upregulation of the endothelial-specific marker CD31 and enhanced expression of VEGFA and FGF-2 and their corresponding receptors PDGFRα and FGFR2, respectively.

**Conclusions:**

A novel eNOS-expressing minicircle vector can efficiently transfect rBMSCs and produce sufficient NO to enhance in vitro models of capillary formation and cell migration with an accompanying upregulation of CD31, angiogenic growth factor, and receptor gene expression.

## Background

Development of safe and efficient systems for gene transfer is required for translation of gene-modified stem cells into therapeutic applications. Conventional plasmid DNA (pDNA)-based non-viral vectors contain bacterial sequences and transcriptional units that may contribute to an immune response against bacterial proteins expressed from cryptic upstream eukaryotic expression signals. Furthermore, changes in eukaryotic gene expression may be altered due to the antibiotic resistance marker and immune responses to bacterial CpG sequences [1]. These prokaryotic DNA sequences present in pDNA vectors may lower their biocompatibility and safety. In clinical studies, un-methylated CpG motifs induced inflammatory responses [2] and necrosis- or apoptosis-mediated cell death in target cells, resulting in short-lived transgene expression [3, 4]. Furthermore, during the intracellular trafficking of pDNA, the bacterial sequences of pDNA vectors are rapidly associated with histone proteins, packing the sequences into a dense heterochromatin structure. If these are spread into the adjacent transgene in the vector, the sequences can become inaccessible by transcription factors, leading to reduced transgene expression through silencing of the eukaryotic promoter [5]. The removal of CpG islands by cloning out, or elimination of non-essential sequences, can reduce these undesirable responses but is time-consuming and tedious.

Minicircle (MC) pDNA technology consists of supercoiled DNA molecules for non-viral gene transfer, which has neither a bacterial origin of replication nor an antibiotic resistance gene [6]. MCs can be generated in *E. coli* ZYCY10P3S2T by attachment sites ((*att*P and *att*B), with specific recombination mediated by the phage ΦC31 integrase [1]. As a result of this recombination event between *att*P and *att*B sites, MCs contain only a eukaryotic expression cassette and the *att*R fragments are formed but are devoid of bacterial backbone sequences. Absence of the bacterial backbone sequences leads to a size reduction in the MC relative to the parental pDNA which can enhance in vitro transfection efficiency [7] and in vivo gene delivery [8, 9]. Gene expression from non-viral episomal vectors may also enhance persistence of transgene expression without interrupting to the cellular genome [10].

Endothelial nitric oxide synthase (eNOS), also known as NOS3, is expressed in endothelial cells [11], and is responsible for generating nitric oxide (NO) which plays an important role in vasculogenesis [12, 13]. NO produced from endothelial cells is important for maintaining vascular integrity and may enhance vasculogenesis through fibroblast growth factor (FGF) signalling [14]. Vascular endothelial growth factor (VEGF) is also induced by the NO synthesis pathway [15] contributing to angiogenesis. eNOS knockout mice (eNOS^−/−^) display impaired vasculogenesis [16] and have also demonstrated diminished wound healing due to reduced VEGF-mediated migration of endothelial cells [17] and bone marrow progenitor cells [18] to the sites of injury. eNOS-based gene therapy approaches have shown restoration of impaired angiogenesis in rats [19, 20] and promotion of re-endothelialisation [21] in injured rabbits upon adenovirus-mediated eNOS gene transfer.

Similar to endothelial progenitor cells, mesenchymal stem cells (MSCs) also participate in post-natal angiogenesis [22], and vascular pericytes, which are crucial for maintaining vascular integrity, share similar phenotypic features with MSCs [23]. Exogenously administered, MSCs form new capillaries and medium-sized arteries [24] which are important properties of tissue regeneration by MSCs [25]. MSCs can differentiate into endothelial cells in vitro [26] and contribute to neovascularisation, particularly during tissue ischaemia and tumour vascularisation [27]. In MSCs, VEGF-A binds with platelet-derived growth factor receptor (PDGFR) to initiate VEGF-A/PDGFR signalling and drive vasculogenesis, as opposed to the VEGFR2 in endothelial cells, which is absent on MSCs [28]. NO has been shown to upregulate PDGFRα receptor expression in rat mesangial cells [29], and the induction of tumour angiogenesis has been linked to the NO-induced Notch signalling pathway in PDGFR-activated mouse glioma cells [30]. FGF2 signalling also enhances vasculogenesis through promotion of NO production [31, 32]. eNOS is the only NOS isoform absent in MSCs [13], and hence eNOS-based genetic modification of MSCs may enhance their therapeutic application. In this study, we describe a novel non-viral MC vector to deliver the eNOS transgene to MSCs with higher transfection efficiency than regular plasmids. NO signalling in the gene-modified MSC promotes capillary tube-like network formation and cell motility. Quantitative real time polymerase chain reaction (PCR) data revealed that MC-mediated eNOS gene transfer significantly upregulates endothelial-specific CD31 gene expression. Furthermore, NO upregulates the angiogenic responsive genes VEGF-A and FGF2 and expression of their corresponding receptors, PDGFRα and FGFR2.

## Methods

### Rat bone marrow-derived mesenchymal stem cell isolation

All experiments involving animals were approved by the Charles Sturt University animal ethics committee. MSCs were isolated from the bone marrow of 8–12 week old male Sprague–Dawley rats as previously described [33].

### Tri-lineage differentiation of rat bone marrow-derived mesenchymal stem cells

The ability of the isolated rat bone marrow-derived MSCs (rBMSCs) (Passage 6) to differentiate to adipogenic, osteogenic and chondrogenic lineages was investigated. To induce osteogenic differentiation, rBMSCs at 80–90 % confluency were incubated in osteogenic-defined medium (Dulbecco’s modified Eagle medium (DMEM) supplemented with 10 % fetal bovine serum (FBS), 10 mM beta-glycerol phosphate, 10 nM dexamethasone and 0.2 mM L-ascorbic acid 2-phosphate) for 11 days with medium changed twice a week, as described previously [34]. Cells were then fixed with 4 % paraformaldehyde and stained with Alizarin Red S (pH 4.1) as described previously [35].

To induce adipogenic differentiation, rBMSCs at 80–90 % confluency were incubated in adipogenic-defined medium (DMEM supplemented with 10 % FBS, 10 μM indomethacin, 1 μM dexamethasone, 0.8 μM insulin, 0.5 mM rosiglitazone) [36] for 1 week with media changed twice. Adipogenic differentiation was assessed by 0.18 % Oil Red O staining after fixing the cells in 10 % neutral-buffered formalin (NBF) [35].

To induce chondrogenic differentiation, three-dimensional pellet cultures of rBMSCs (2.5 × 10^5^ cells) were formed by centrifugation at 500 × *g* in 10 ml conical-bottomed sterile tubes. The chondrogenic induction medium consisted of DMEM supplemented with 1 × ITS + 3 (Sigma), 1 × non-essential amino acids (Sigma), 10 ng/ml transforming growth factor β (TGF-β3; Peprotech), 100 nM dexamethasone, and 2 μM ascorbic acid (Sigma) [37]. Pellet cultures were incubated in induction medium for 14 days with the medium changed every second day with the lids of the tube loosened to facilitate gas exchange. At day 14 the pellets were fixed in 10 % NBF for 24 h, and the three-dimensional tissues were processed and embedded in paraffin wax for microtome processing. To assess chondrogenic differentiation, embedded pellets were sectioned (5 μm slices) and stained with 1 % Alcian blue to visualise glycosaminoglycan accumulation.

The images for differentiated cells into all three lineages were captured by a colour camera (Nikon Digital Sight Ds-Fi2) attached to a Nikon Eclipse-Ti-U microscope (Nikon).

### Production of minicircle plasmid DNA-expressing eNOS

To construct an eNOS expressing minicircle vector, a codon optimized human eNOS cDNA sequence (3633 bp) was cloned into the minicircle parental plasmid consisting of expression cassette CMV–MCS–EF1α–GFP–SV40–PolyA (P-GFP) (System Biosciences, Mountain View, CA, USA). This cloning strategy allowed removal of the EF1α–GFP portion from the final construct (P-eNOS).

The minicircle DNA plasmids expressing eNOS and GFP were produced according to the manufacturer’s instructions (System Biosciences). Briefly, *E. coli* ZYCY10P3S2T cells were transformed with P-GFP and P-eNOS. Following this, single colonies were grown in 2 ml LB (luria broth) media containing 50 μg/ml kanamycin for 1 h at 30 °C with vigorous shaking at 200 rpm. Next, 50 μl of the starter culture was then used to inoculate 200 ml fresh terrific broth (TB; Sigma) in a 1 litre flask with 50 μg/ml kanamycin followed by incubation at 30 °C for 17 h with constant shaking at 200 rpm. Minicircle induction medium consisting of 200 ml LB (luria broth), 8 ml 1 N NaOH and 200 μl 20 % L-arabinose was combined with the TB bacterial culture and incubated for a further 4 h at 30 °C with constant shaking at 200 rpm. Minicircle plasmid DNA (MC-eNOS and MC-GFP) was isolated using a Genomed Jetstar 2.0 midi kit according to the manufacturer’s instructions (Genomed, Germany) and treated with plasmid-safe ATP-dependent DNase (Epicentre, USA) to remove bacterial genomic DNA contamination. eNOS- and GFP-containing minicircles were designated as MC-eNOS and MC-GFP, respectively.

### Cell culture and transfection

Human embryonic kidney (HEK293T) cells and rBMSCs were maintained in DMEM (Sigma) supplemented with 10 % (v/v) FBS (Sigma), 1 % (v/v) L-glutamate (Sigma) and 1 % (v/v) penicillin/streptomycin antibiotics mix (Sigma). Cells were transfected with the plasmids (P-GFP, MC-GFP, P-eNOS and MC-eNOS) using Lipofectamine 2000 reagent (Life technologies, USA) following the manufacturer’s instructions. GFP expression was assessed by fluorescence microscopy at 24 and 48 h after transfection, and flow cytometry analysis (Gallios Instrument, Beckmann).

### Immunocytochemistry

Immunocytochemical detection of eNOS expression in P-eNOS and MC-eNOS transfected HEK293T and rBMSCs was performed as follows. Briefly, cells were fixed in 4 % paraformaldehyde for 20 min at room temperature, treated with 0.1% Triton-X100 in phosphate-buffered saline (PBS) for 10 min, and blocked in a 10 % FBS in PBS solution for 30 min at room temperature. This was followed by a 2-h incubation with a primary mouse monoclonal anti-eNOS antibody (BD Bioscience), and subsequently with an anti-mouse IgG secondary antibody conjugated with Alexa 488 (Cell Signalling Technology) for 1 h followed by DAPI (nuclear stain) and phalloidin-TRITC (cytoskeleton stain) (Sigma). eNOS-positive cells were counted by fluorescence microscopy in five randomly selected fields per well in three independent experiments and 500–1000 cells were counted in total; the percentage of eNOS positivity was calculated from the total nuclear stained cells.

### Nitric oxide detection

Nitric oxide released from P-eNOS and MC-eNOS transfected cells in cell supernatants was measured using the griess reagent (Promega) following the manufacturer’s instructions. NO was also directly detected in transfected cells using a specific fluorescent NO indicator, 4,5-diaminofluorescein diacetate (DAF-2DA; Cayman chemicals, USA), as described previously [13, 38]. Cells were grown to confluence on a 12-well plate and incubated for 30 min with 1 μM DAF-2DA. Subsequently, cells were washed with fresh PBS and viewed by a fluorescence microscope.

### In vitro angiogenesis

In vitro capillary formation was performed as described previously [39]. Briefly, Geltrex™ (Life technologies) was thawed on ice overnight and applied evenly over each well (50 μl) of a 96-well plate and incubated for 30 min at 37 °C allowing polymerisation. Transfected rBMSCs or control cells were seeded at 20,000 cells per well and grown in 100 μl angiogenic induction medium (DMEM (Sigma), 1.5 % FBS, 1 % (v/v) L-glutamate (Sigma) and 1 % (v/v) penicillin/streptomycin (Sigma)) and incubated at 37 °C for 5 h. The capillary network was fixed with 4 % paraformaldehyde and visualized by staining with DAPI and Phalloidin (Sigma). The efficiency of in vitro tubule formation was evaluated by measuring the number of nodes and length of the tubules as described previously [13].

### In vitro scratch wound healing assay

The effect of nitric oxide on cell migration was assessed using an in vitro scratch wound healing assay as described previously [37]. Briefly, HEK293T cells and rBMSCs were transfected with P-eNOS, MC-eNOS, P-GFP and MC-GFP in 6-well tissue culture plates. Next, 48 h following the transfection when the cells reached 100 % confluence, scratch wounds were made using a sterile 200 μl pipette tip and the boundaries were marked. The cells were then cultured with 2 ml fresh DMEM supplemented with 10 % (v/v) FBS (Sigma), 1 % (v/v), L-glutamate (Sigma), and 1 % (v/v) penicillin/streptomycin (Sigma). Phase-contrast microscopy images were acquired at 0 and 1 h after scratches were created for rBMSCs and after17 h for HEK293T cells. Cell migration was measured at the indicated times by measuring the distance from the initial boundary edge to the boundary of the migrating cells, followed by calculation of the percentage of wound closure as follows: percentage of wound closure = (distance from the boundary edge at 0 h – distance from the boundary edge at 1 h or 17 h)/(distance from the boundary edge at 0 h) × 100.

### Gene expression by quantitative real time PCR

Total RNA from transfected and control cells was isolated using the PureZol reagent (BioRad) according to the manufacturer’s instructions and the concentration of isolated RNA was determined using a Nanodrop spectrophotometer (Thermo Scientific) following treatment with RQ1 RNase free DNase (Promega) to remove contaminating DNA. Then, cDNA was synthesized with 1 μg RNA using a High Capacity Reverse Transcription Kit (Life technologies). The quantitative real time PCR assays were performed on a BioRad CFX96 Real-Time system (BioRad) using the SsoFast EvaGreen Supermix (BioRad). Primers used for target amplification are described in Table 1. Assays were performed in triplicate, and target mRNA expression was normalized to rat GAPDH mRNA levels using the ΔCt method.

**Table 1:** Primers used in this study

Target	Forward	Reverse	Expected size	Accession number
VEGF-A	GGTGGACATCTTCCAGGAGT	TGATCTGCATGGTGATGTTG	146	NM_001317043
FGF2	GCTGCTGGCTTCTAAGTGTG	TACTGCCCAGTTCGTTTCAG	129	NM_019305
PDGFR α	TTGAGCCCATTACTGTTGGA	CCCATAGCTCCTGAGACCTT	148	NM_011058
FGFR2	GACGACACAGATAGCTCCGA	CAGCGGAACTTCACAGTGTT	134	EF143338
CD31	CATTGGTTACCTCGGGAGTC	GTCTTCACCCAGCCTTTCTC	104	NM_001107202
GAPDH	ACAGCAACAGGGTGGTGGAC	TTTGAGGGTGCAGCAACTT	252	NM_017008.4

### Western blot analysis

Transfected and control cells were washed with ice-cold PBS (Sigma) twice, and lysates were prepared by homogenization of cells in RIPA buffer (Sigma), following mixing with 4 × NuPAGE LDS sample buffer (Life technologies) and lysed by heating for 10 min at 70 °C. Total proteins were separated by 4–12 % Bis-Tris NuPAGE (Novex, Life technologies) and transferred to PVDF membrane (Millipore). After blocking with odyssey blocking buffer (LI-COR) for 30 min at room temperature, the membrane was incubated with primary antibodies specific to eNOS (1:1000 dilution) and β-actin (LI-COR; 1:1000 dilution) overnight at 4 °C. The membrane was washed with 0.1 % tween in PBS three times for 10 min each, incubated with donkey anti-rabbit IgG (H&L) (Alexa Fluor® 680) secondary antibody (Life technologies; 1:20,000) at room temperature for 1 h, and antibody-bound proteins were visualized by fluorescence detection with a LI-COR odyssey system.

### Statistical analysis

All experiments were performed in triplicate and at least three times and data analysed by an independent two-tailed Student’s *t* test. A *p* value <0.05 was regarded as statistically significant.

## Results

### Characterisation of rBMSCs

**Fig. 1:**
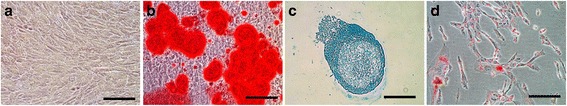
Characterization of rBMSCs. Tri-lineage differentiation of rBMSC was performed in vitro. **a** Undifferentiated rBMSC. **b** Alizarin red S staining of cells cultured for 14 days in osteogenic induction medium. **c** Alcian blue staining and toluidine blue staining of cells cultured for 14 days in chondrogenic induction medium. **d** Oil red O staining of cells cultured for 7 days in adipogenic induction medium. *Scale bar* = 100 μm

rBMSCs were isolated from adult Sprague–Dawley rats as previously described [33], and plastic adherent rBMSCs displayed typical fibroblastoid morphology (Fig. 1a) [40]. Tri-lineage differentiation of the rBMSCs was performed in the appropriate media to osteoblasts as demonstrated by Alzarin Red S staining of mineralised extracellular matrix (Fig. 1b), to chondrocytes by Alcian Blue staining of proteoglycans in three-dimensional pellet cultures (Fig. 1c) and to adipocytes as shown by Oil Red O staining of lipid vesicles (Fig. 1d).

### Transfection of P-GFP and MC-GFP vectors

**Fig. 2:**
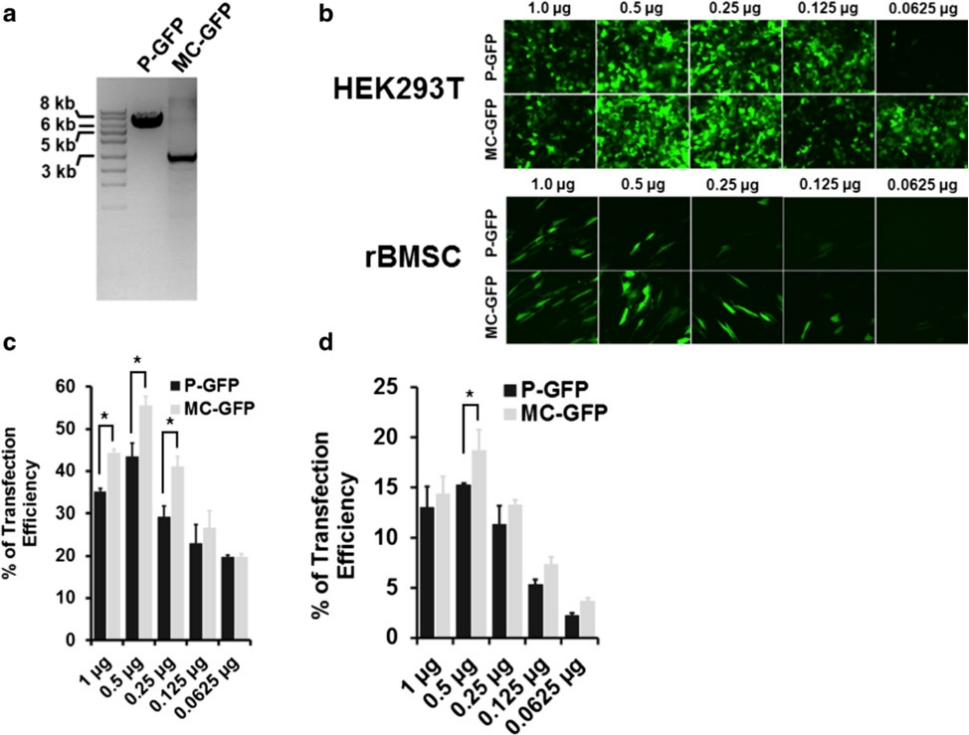
Gene delivery efficiency of P-GFP and MC-GFP DNA vectors. **a** Gel electrophoresis of P-GFP and MC-GFP plasmids following plasmid purification and enzyme digestion. **b** Fluorescence microscopy of transfected HEK293T cells and rBMSCs with P-GFP and MC-GFP with a range of plasmid concentrations and quantitation by flow cytometry of transfection efficiencies for (**c**) HEK293T cells and (**d**) rBMSCs. *MC-GFP* minicircle vector expressing green fluorescent protein, *P-GFP* plasmid vector expressing green fluorescent protein, *rBMSC* rat bone marrow-derived mesenchymal stem cell

The GFP expressing minicircle vector (MC-GFP) was produced from the parental plasmid (P-GFP) as described in the manufacturer’s instructions (Systems Bioscience). We observed an approximate 4-kb reduction in plasmid size following minicircle induction using L-arabinose (Fig. 2a). HEK293T cells and rBMSCs were transfected with a range of plasmid DNA concentrations (1 μg, 0.5 μg, 0.25 μg, 0.125 μg, 0.0625 μg,). After 48 h post-transfection, the cells were visualised by fluorescence microscopy and analysed by flow cytometry to estimate the percentage of GFP-expressing (GFP+) cells (Fig. 2b). The optimum plasmid DNA concentration for transection was 0.5 μg which showed highest transfection efficiency for both P-GFP and MC-GFP in both HEK293T (Fig. 2c) and rBMSC (Fig. 2d) cell types.

Transfection of HEK293T cells with MC-GFP plasmid resulted in a significantly higher number of GFP+ cells ((55.51 ± 3.3 %) compared to P-GFP (43.4 ± 4.9 %). A similar trend was seen in rBMSCs, with MC-GFP resulting in a higher transfection efficiency (18.65 ± 1.05 %) compared to P-GFP (15.21 ± 0.22 %).

### Generation of eNOS minicircle vector

**Fig. 3:**
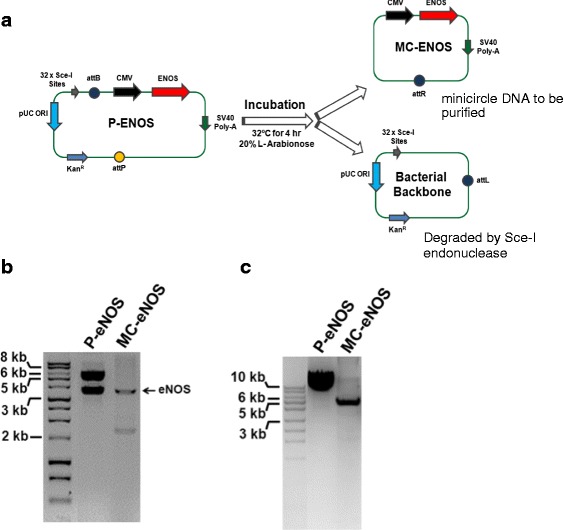
Construction of eNOS expressing minicircle DNA vector. **a** Schematic representation of in vitro production of MC-eNOS vector. **b** Confirmation of cloning of eNOS gene into P-eNOS and MC-eNOS vectors by restriction enzyme digestion analysis. **c** P-eNOS and MC-eNOS gel electrophoresis following minicircle plasmid purification. *MC-eNOS* minicircle vector expressing endothelial nitric oxide synthase, *P-eNOS* plasmid vector expressing endothelial nitric oxide synthase

To generate an eNOS minicircle expression plasmid vector, a codon optimized cDNA of human eNOS (3633 bp) was synthesised (Geneart) and sub-cloned into the parental plasmid P-GFP (CMV-MCS-EF1-GFP-SV40PolyA) (System Biosciences, Mountain View, CA, USA) at the *Bam*HI and *Sal*I restriction sites in the multiple cloning sites downstream to the CMV promoter resulting in removal of the EF1α promoter and eGFP coding sequence (Fig. 3a). The eNOS minicircle vector was constructed as described above for the MC-GFP vector. The cloning was confirmed by double digestion of the parental plasmid encoding eNOS (P-eNOS) with *Bam*HI and *Sal*I yielding a fragment of ~3.7 kb (Fig. 3b). A reduction of the P-eNOS vector size was also observed after the production of MC-eNOS, to approximately 5 kb (Fig. 3c).

### Transfection of P-eNOS and MC-eNOS vectors

**Fig. 4:**
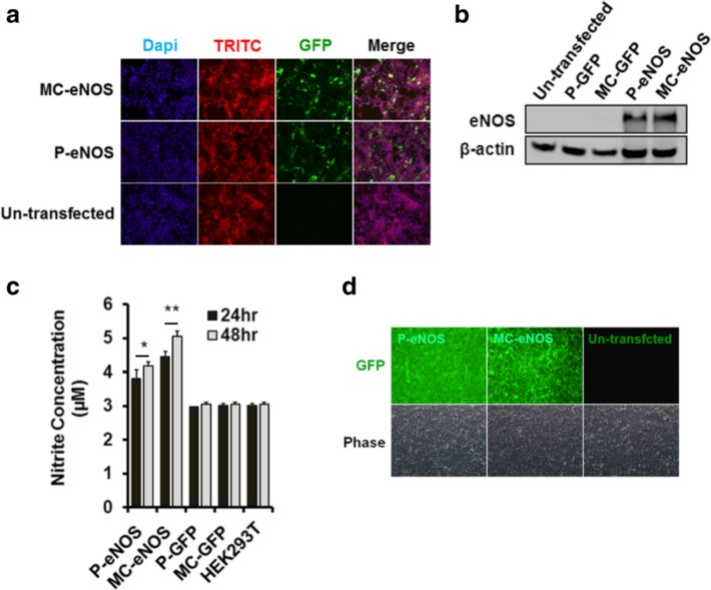
Expression of eNOS and NO production in transfected HEK293T cells. **a** Fluorescence microscopy of transfected HEK293T cells with P-eNOS and MC-eNOS with 0.5 μg plasmid DNA. **b** Detection of eNOS protein expression in transfected HEK293T by western blot analysis. **c** NO production in HEK293T cells at 24 h and 48 h post-transfection with P-eNOS and MC-eNOS plasmids using the griess assay, and **d** detection of nitric oxide production in living cells following P-eNOS and MC-eNOS transfection and non-transfected control by DAF-2 fluorescence. *p < 0.05 and **p < 0.05 vs. MC-GFP, P-GFP, and HEK293T. *MC-eNOS* minicircle vector expressing endothelial nitric oxide synthase, *MC-GFP* minicircle vector expressing green fluorescent protein, *P-eNOS* plasmid vector expressing endothelial nitric oxide synthase, *P-GFP* plasmid vector expressing green fluorescent protein

Transfection of HEK293T cells with P-eNOS and MC-eNOS was assessed by immunofluorescence staining (Fig. 4a) and western blot analysis (Fig. 4b), using an eNOS-specific monoclonal antibody (BD bioscience). Nitric oxide production from transfected cells was measured by the production of nitrite at 24 hand 48 h post-transfection and in un-transfected HEK293T cells (Fig. 4c). Both P-eNOS and MC-eNOS transfected HEK293T cells showed significantly higher nitrite accumulation in cell culture media (at both 24 h and 48 h) compared to P-GFP, MC-GFP transfected cells and un-transfected HEK293T controls. At 24 h post-transfection, HEK293T cells transfected with P-eNOS and MC-eNOS resulted in 3.8 ± 0.2 μM and 4.46 ± 0.12 μM nitrite concentrations, respectively (Fig. 4c). The NO production increased significantly at 48 h post-transfection, resulting in 4.18 ± 0.12 μM and 5.06 ± 0.13 μM for P-eNOS and MC-eNOS, respectively. Furthermore, detection of nitric oxide produced from transfected cells was also confirmed by DAF-2DA staining in live cells. Both P-eNOS and MC-eNOS transfected HEK293T cells emitted a strong green fluorescence signal compared to no fluorescence in un-transfected cells (Fig. 4d).

### eNOS gene transfer to rBMSCs

**Fig. 5:**
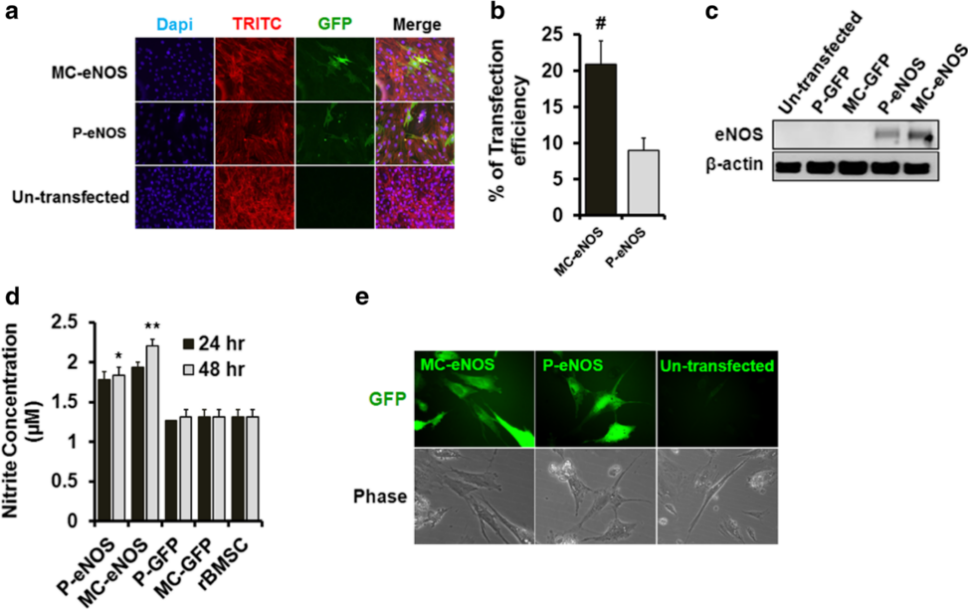
Expression of eNOS and NO production in transfected rBMSCs. **a** Fluorescence microscopy of transfected of rBMSCs with P-eNOS and MC-eNOS with 0.5 μg plasmid DNA. **b** Transfection efficiency of MC-eNOS and P-eNOS. **c** Detection of eNOS protein expression in transfected rBMSC by western blot analysis. **d** NO production in rBMSC cells at 24 hand 48 h post-transfection with P-eNOS and MC-eNOS plasmids using the griess assay. **e** Detection of nitric oxide production in living cells following P-eNOS and MC-eNOS transfection by DAF-2 fluorescence. ^#^ *p* < 0.05 vs. P-eNOS; *p < 0.05 and **p < 0.05 vs. MC-GFP, P-GFP, and rBMSC. *MC-eNOS* minicircle vector expressing endothelial nitric oxide synthase, *MC-GFP* minicircle vector expressing green fluorescent protein, *P-eNOS* plasmid vector expressing endothelial nitric oxide synthase, *P-GFP* plasmid vector expressing green fluorescent protein, *rBMSC* rat bone marrow-derived mesenchymal stem cell

Transfection of P-eNOS and MC-eNOS vectors into rBMSCs was confirmed by immunostaining (Fig. 5a), and western blot analysis (Fig. 5c) with an eNOS-specific monoclonal antibody (BD bioscience). Both the assays confirmed that no endogenous eNOS expression was seen in un-transfected rBMSCs (Fig. 5a and c). Significantly higher transfection efficiency for MC-eNOS (21 ± 3 %) compared to P-eNOS (9 ± 3 %) (Fig. 5b) was observed which resulted in higher NO production for MC-eNOS transfected rBMSCs (1.93 ± 0.06 μM) than P-eNOS (1.78 ± 0.1 μM) (Fig. 5d) compared to controls after 24 h of transfection. NO production increased further in MC-eNOS transfected rBMSCs (2.20 ± 0.08 μM) compared to P-eNOS at 48 h post-transfection (1.84 ± 0.1 μM) (Fig. 5d). NO synthesis in transfected rBMSCs was also demonstrated DAF-2DA staining in both P-eNOS and MC-eNOS transfected rBMSCs (Fig. 5e).

### eNOS gene delivery enhances in vitro capillary tubule formation

**Fig. 6:**
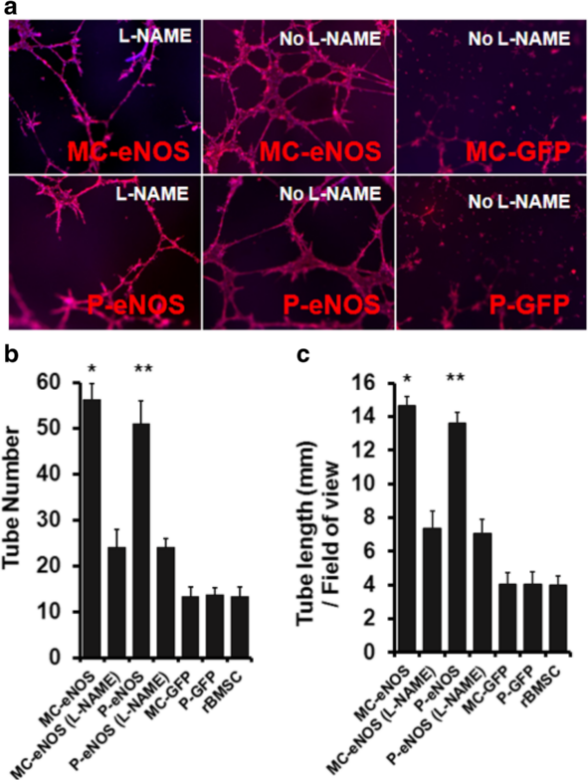
In vitro tubule formation in eNOS transfected rBMSCs. **a** Capillary tubule formation in rBMSCs transfected with P-eNOS and MC-eNOS and cytoskeletal staining by Phalloidin TRITC; treatment with the NO inhibitor L-NAME reduces capillary formation. **b** Quantitation of tubule number. **c** Measurement of tubule length. *p < 0.05 and **p < 0.05 vs. MC-eNOS (L-NAME), P-eNOS (L-NAME), MC-GFP, P-GFP, and rBMSC. *L-NAME*, L-N^G^-nitroarginine methyl ester, *MC-eNOS* minicircle vector expressing endothelial nitric oxide synthase, *MC-GFP* minicircle vector expressing green fluorescent protein, *P-eNOS* plasmid vector expressing endothelial nitric oxide synthase, *P-GFP* plasmid vector expressing green fluorescent protein, *rBMSC* rat bone marrow-derived mesenchymal stem cell

Rat BMSCs were transfected with 0.5 μg P-eNOS, MC-eNOS, P-GFP, and MC-GFP. Un-transfected rBMSCs were used as a control. Transfected cells were then plated on a 96-well cell culture plate coated with an extracellular matrix (Geltrex). Both MC-eNOS and P-eNOS transfected rBMSCs formed significantly longer (14.66 ± 0.55 mm and 13.58 ± 0.68 mm, respectively) tubules and a greater number of tubules (56.33 ± 3.51 and 51 ± 4, respectively) compared to rBMSCs transfected with P-GFP, MC-GFP and non-transfected cells (Fig. 6).

To confirm that capillary-like tubule formation was NO-mediated, eNOS transfected rBMSCs were treated with 2 mM of the nitric oxide synthase inhibitor, L-N^G^-nitroarginine methyl ester (L-NAME). L-NAME treatment resulted in a significant impairment of the tubule network, in terms of length (7.33 ± 1.03 mm and 7.06 ± 0.88 mm for MC-eNOS and P-eNOS, respectively) and tubule number (24 ± 4 and 24 ± 2 for MC-eNOS and P-eNOS, respectively) compared to untreated cells (Fig. 6).

### Nitric oxide promotes in vitro cell migration

**Fig. 7:**
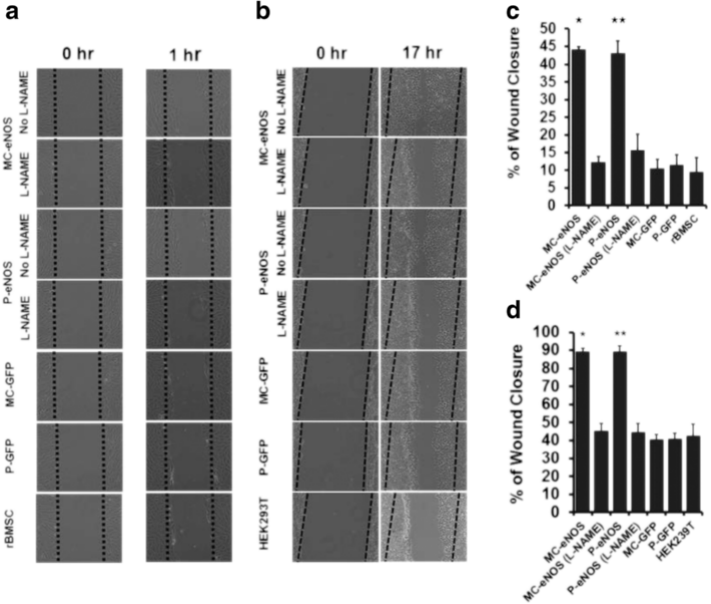
In vitro cell scratch assay of eNOS transfected rBMSCs and HEK293T cells. Phase-contrast microscopy images of transfected and control cell migration at (**a**) 0 and 1 h for rBMSCs and (**b**) 0 and 17 h for HEK293T post-cell scratch and effect of the NO inhibitor L-NAME. Percentage of cell migration (**c**) at 1 h for rBMSCs and (**d**) 17 h for HEK293T post-cell scratch and effect of the NO inhibitor L-NAME. *p < 0.05 and **p < 0.05 vs. MC-eNOS (L-NAME), P-eNOS (L-NAME), MC-GFP, P-GFP, rBMSC and HEK293T. *L-NAME*, L-N^G^-nitroarginine methyl ester, *MC-eNOS* minicircle vector expressing endothelial nitric oxide synthase, *MC-GFP* minicircle vector expressing green fluorescent protein, *P-eNOS* plasmid vector expressing endothelial nitric oxide synthase, *P-GFP* plasmid vector expressing green fluorescent protein, *rBMSC* rat bone marrow-derived mesenchymal stem cell

Using the scratch wound healing assay [37], migration of eNOS transfected rBMSCs was assessed. Transfection of P-eNOS and MC-eNOS enhanced cell migration compared to P-GFP, MC-GFP and un-transfected rBMSCs (MC-eNOS, 44.05 ± 0.81 %; P-eNOS, 43.13 ± 3.45 %; MC-GFP, 10.43 ± 2.63 %; P-GFP, 11.39 ± 3.03 %; and rMSC, 9.46 ± 4.13 %) (Fig. 7a and c). However, cell migration rates between P-eNOS and MC-eNOS were not significantly different. Inhibition of NO production by treatment with 2 mM L-NAME significantly diminished the cell migration rates of both MC-eNOS and P-eNOS transfected cells (12.18 ± 1.67 % and 15.59 ± 4.69 %, respectively) (Fig. 7a and c). Cell migration rates were not significantly different among MC-GFP, P-GFP and un-transfected cells (Fig. 7a and c). A similar phenomenon was observed with HEK293T cells (Fig. 7b and d).

### MC-eNOS gene transfer to rBMSCs induces endothelial CD31 gene expression

**Fig. 8:**
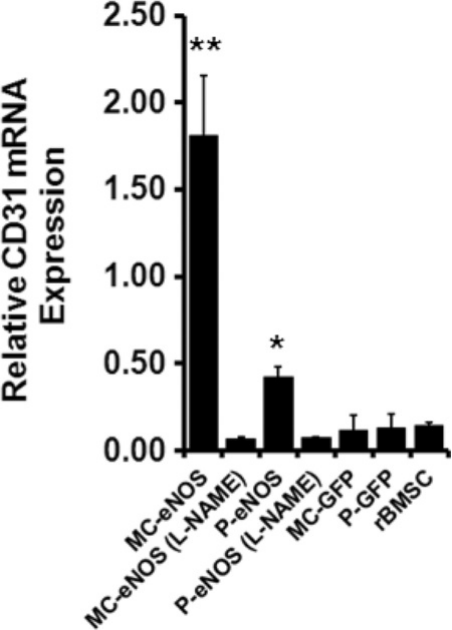
Nitric oxide promotes CD31 gene expression in eNOS transfected rBMSCs. Relative mRNA expression of endothelial-specific CD31 was upregulated in MC-eNOS and P-eNOS transfected rBMSCs as assessed by quantitative real time PCR. *p < 0.05 and **p < 0.05 vs. MC-eNOS (L-NAME), P-eNOS (L-NAME), MC-GFP, P-GFP, and rBMSC. *L-NAME*, L-N^G^-nitroarginine methyl ester, *MC-eNOS* minicircle vector expressing endothelial nitric oxide synthase, *MC-GFP* minicircle vector expressing green fluorescent protein, *P-eNOS* plasmid vector expressing endothelial nitric oxide synthase, *P-GFP* plasmid vector expressing green fluorescent protein, *rBMSC* rat bone marrow-derived mesenchymal stem cell

We found a significant increase in CD31 mRNA expression by 0.42-fold in P-eNOS transfected cells compared to P-GFP, MC-GFP and un-transfected control, suggesting that eNOS gene transfer may promote endothelial differentiation of rBMSCs (Fig. 8). Interestingly, minicircle-mediated eNOS (MC-eNOS) gene transfer showed a highly significant increase in CD31 mRNA expression by 1.8-fold compared to P-eNOS, P-GFP, MC-GFP, and un-transfected control (Fig. 8). Treatment with 2 mM L-NAME abolished the CD31 expression (Fig. 8), suggesting that expression of endothelial CD31 in rBMSCs through eNOS gene transfer is NO-mediated.

### NO modulates VEGF-A/PDGFR and FGF2/FGFR2 signalling pathways in eNOS transfected rBMSCs

**Fig. 9:**
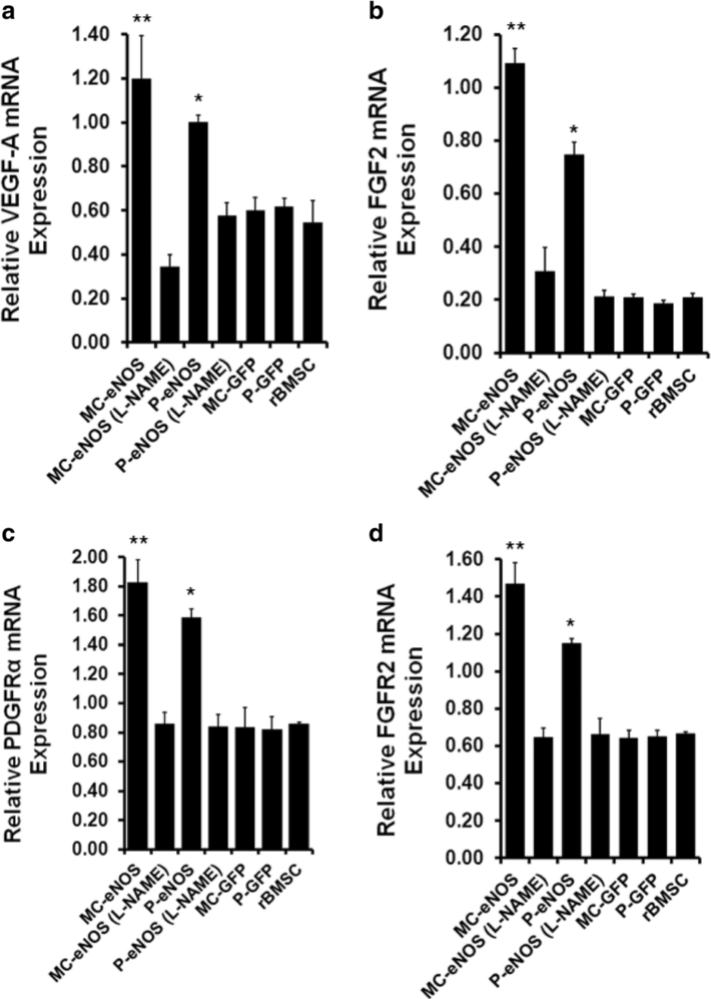
Nitric oxide modulates VEGF-A/PDGFRα and FGF2/FGFR2 gene expression. Relative mRNA expression of the angiogenesis-related genes (**a**) VEGF-A and (**b**) FGF2 and their corresponding receptors, **c** PDGFRα and **d** FGFR2, were upregulated in MC-eNOS and P-eNOS transfected rBMSCs as assessed by quantitative real time PCR. *p < 0.05 and **p < 0.05 vs. MC-eNOS (L-NAME), P-eNOS (L-NAME), MC-GFP, P-GFP, and rBMSC. *FGF(R)* fibroblast growth factor (receptor), *L-NAME*, L-N^G^-nitroarginine methyl ester, *MC-eNOS* minicircle vector expressing endothelial nitric oxide synthase, *MC-GFP* minicircle vector expressing green fluorescent protein, *PDGFR* platelet-derived growth factor receptor, *P-eNOS* plasmid vector expressing endothelial nitric oxide synthase, *P-GFP* plasmid vector expressing green fluorescent protein, *rBMSC* rat bone marrow-derived mesenchymal stem cell, *VEGF* vascular endothelial growth factor

Expression of two key genes, VEGF-A and FGF2, which are involved in angiogenesis and cell migration were examined by quantitative real time PCR. Upregulation of both VEGF-A by 1.19-fold and 1.0-fold in MC-eNOS and P-eNOS modified rBMSCs, respectively (Fig. 9a), and FGF2 by 1.08-fold in MC-eNOS and 0.74-fold in P-eNOS delivered rBMSCs (Fig. 9b), compared to P-GFP, MC-GFP delivered rBMSCs and un-transfected rBMSCs. Treatment with 2 mM L-NAME reduced both VEGF-A (Fig. 9a) and FGF2 (Fig. 9b) expression in P-eNOS and MC-eNOS transfected cells. Furthermore, delivery of P-GFP and MC-GFP did not affect the VEGF-A and FGF2 expression compared to control rBMSCs (Fig. 9a and 9b). Next, we examined the effect of NO on the expression of PDGFRα and FGFR2 receptors as they are corresponding receptors of VEGF-A and FGF2. Expression of PDGFRα was increased by 1.82-fold and 1.56-fold in MC-eNOS and P-eNOS transfected rBMSCs, respectively (Fig. 9c), and FGFR2 receptor expression was increased by 1.46-fold in MC-eNOS and 1.14-fold in P-eNOS delivered rBMSCs (Fig. 9d), compared to P-GFP, MC-GFP delivered rBMSCs and un-transfected rBMSCs. Treatment with 2 mM L-NAME abolished both the PDGFRα (Fig. 9c) and FGFR2 (Fig. 9d) expression in P-eNOS and MC-eNOS transfected cells. Furthermore, neither PDGFRα nor FGFR2 receptor expression were affected by the delivery of P-GFP and MC-GFP compared to control rBMSCs (Fig. 9c and d).

## Discussion

Minicircle vectors are supercoiled DNA molecules that are devoid of bacterial backbone sequences such as a bacterial origin of replication, antibiotic resistance gene and CpG motifs [41], and primarily consist of a eukaryotic expression cassette [6]. Compared to conventional plasmid DNA, minicircle vectors benefit from higher transfection efficiencies and longer transgene expression, possibly attributed to a lower activation of gene silencing mechanisms [42].

In this study, minicircles expressing GFP exhibited higher in vitro gene transfer efficiency than the parental plasmid to both HEK293T cells and rBMSCs (Fig. 3). As expected transfection efficiency was higher in the transformed cell line (HEK293T) compared to primary rBMSCs. eNOS expressing minicircles also showed higher gene transfer efficiency than P-eNOS (Fig. 5b). This higher gene transfer efficiency may also account for the significantly increased level of NO synthesis by MC-eNOS compared to P-eNOS (Figs. 4 and 5). We reasoned that this high level of NO synthesis from MC-eNOS transfected rBMSCs may be attributed to the removal of other plasmid sequences, which can affect gene expression [42], and the smaller size of the minicircle may also provide a more efficient route to the nucleus for transcription. This process involves several steps, including cellular entry of DNA through the cell membrane, DNA diffusion into the cytoplasm, and DNA entry to the nucleus [43]. Importantly, the DNA diffusion step depends on the physicochemical properties of DNA such as its diffusion coefficient, which is inversely proportional to its molecular weight [44, 45]. Endocytosis is a major route for entry of DNA–cationic lipid complexes through the cell membrane in vitro [46] which takes place following specific interactions between DNA and caveolae [47]. This mechanism is also limited by particle size, where larger DNA–cationic lipid complexes are not efficiently taken up by the endocytosis [47]. DNA uptake and transfer to the nucleus via the nuclear membrane results in successful gene transfer [48]. Minicircle plasmid vector may overcome these cellular obstacles more efficiently and, combined with a lack of bacterial backbone sequences, reduced promoter methylation [49] may also contribute to the higher levels of gene transfer compared to larger parental plasmids.

Angiogenesis is a complex process involving endothelial cell proliferation and migration, remodelling of extracellular matrix, and tubular structure formation. These processes are tightly regulated by the actions of angiogenic cytokines such as VEGF-A and FGF [31]. Angiogenesis also requires endothelial cell-to-cell, and cell-to-matrix interactions, which are mediated by various cell adhesion molecules [50]. eNOS plays a key role in angiogenesis mediated by substance P, a potent endothelium-dependent vasodilator (NO releaser) [51]. It has also been demonstrated that eNOS-KO (knockout) mice show impaired angiogenesis [52].

NO has been shown to play an important role in angiogenesis both in vitro and in vivo, and furthermore NO also contributes to endothelial cell migration in vitro [52]. We found that eNOS gene transfer by MC vector remarkably promoted endothelial-specific CD31 gene expression (Fig. 8), contributing to the capillary-like tubule network formation by rBMSCs (Fig. 6) and enhanced cell motility as evident by in vitro wound healing assay (Fig. 7). Noteworthy, in these assays, CD31 mRNA expression, tubule formation and cell migration in transfected cells were significantly abrogated by L-NAME treatment, suggesting NO plays a major role in enhancing endothelial characteristics in rBMSCs. Collectively, our data may suggest that MC-mediated eNOS gene transfer may contribute to the reprogramming of adults stem cells into endothelial cells, which may be used in cell therapy applications involving vascular repair. Interestingly, Gomes and co-workers demonstrated that MSCs from S-nitrosoglutathione reductase (GSNOR)-deficient mice, where NO is produced mainly from iNOS (NOS2) rather than eNOS, exhibited attenuated vasculogenesis both in vitro and in vivo [13]. Furthermore, they revealed that pharmacological inhibition of NO in GSNOR^−/−^ MSCs, or genetic reduction of NO production in the NOS2^−/−^, enhanced vasculogenesis by MSCs than that for HUVECs, where NO synthesis is driven by eNOS enhanced vascular tube formation. MSCs have not been shown to express endogenous eNOS, unlike endothelial cells [53], and have been shown to participate in pro-angiogenic signalling [54]. Additionally, eNOS plays an important role in endothelial cell-mediated postnatal angiogenesis and vascular tone [55, 56].

NO may contribute to angiogenesis through VEGF and FGF signalling through an angiogenic switch which is preceded by a local increase in VEGF-A and FGF [31]. Nitric oxide can mediate the production of VEGF-A in human adipose-derived stem cells [57] and NO and FGF2 have also been shown to enhance angiogenesis in mouse embryonic stem cells [58]. Furthermore, FGF2 has been shown to induce eNOS expression [32]. Our data proposes that NO signalling through VEGF-A/PDGFRα and FGF2/FGFR2 pathways may directly promote rBMSC vasculogenesis (Fig. 9). We showed that eNOS transfected rBMSCs express increased levels of VEGF-A and FGF2 (Fig. 9) and their corresponding receptors PDGFRα, and FGFR2, respectively (Fig. 9). It is noteworthy that MC-eNOS vector transfection was associated with a significantly higher FGF2 expression compared to the P-eNOS vector. Interestingly, treatment with L-NAME diminished the VEGF-A, PDGFRα, FGF2 and FGFR2 expression levels (Fig. 9) which were observed as being linked to impaired capillary tube-like network formation (Fig. 6). It has been shown that VEGF-A contributes to differentiation of MSCs to endothelial-like cells when co-cultured with endothelial cells expressing eNOS and this process is inhibited by VEGF-A antisera [59].

**Fig. 10:**
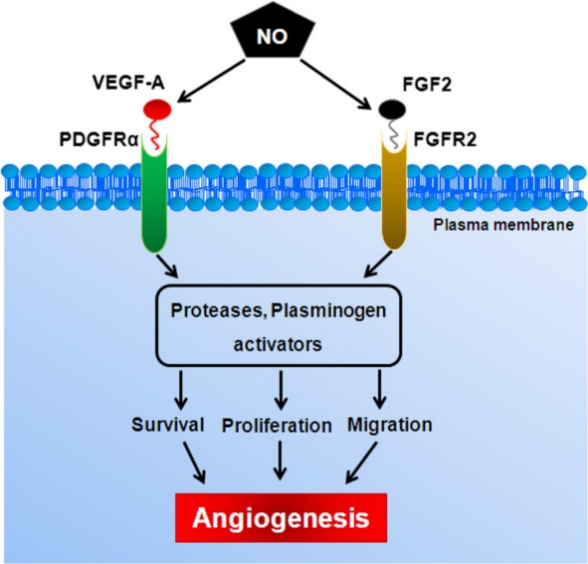
Proposed molecular mechanism underlying the NO mediated angiogenic responses by MSCs. *FGF(R)* fibroblast growth factor (receptor), *NO* nitric oxide, *PDGFR* platelet-derived growth factor receptor, *VEGF* vascular endothelial growth factor

Angiogenesis is also associated with endothelial cell migration and proliferation [60]. Our results show that eNOS gene transfer into HEK293T and rBMSCs (Fig. 7) can increase cell motility compared to controls, and the effect is diminished by L-NAME treatment, suggesting that NO plays a role in regulating rBMSC cell migration (Fig. 7) which has been previously demonstrated for endothelial cell migration [61]. Together, these findings show that genetic manipulation of MSCs to enhance bioavailable NO may upregulate VEGF-A/PDGFRα and FGF2/FGFR2 signalling pathways to promote angiogenesis (Fig. 10).

## Conclusions

In summary, this study demonstrates that NO derived from a minicircle DNA vector expressing eNOS exerts a positive effect on rBMSCs by promoting in vitro capillary tubule formation and cell migration and significant increases in angiogenesis-related gene expression. Use of MC-eNOS-based vectors may represent an efficient approach to gene therapy applications where enhancing NO bioavailability is beneficial.

## Abbreviations

DAF-2DA: 4,5-diaminofluorescein diacetate
DMEM: Dulbecco’s modified Eagle medium
eNOS: endothelial nitric oxide synthase
FBS: fetal bovine serum
FGF(R): fibroblast growth factor (receptor)
GFP: green fluorescent protein
LB: Luria Broth
L-NAME: L-N^G^-nitroarginine methyl ester
MC: minicircle
MSC: mesenchymal stem cell
NBF: neutral-buffered formalin
NO: nitric oxide
PBS: phosphate-buffered saline
PCR: polymerase chain reaction
PDGF(R): platelet-derived growth factor (receptor)
pDNA: plasmid DNA
rBMSC: rat bone marrow-derived mesenchymal stem cell
TB: terrific broth
VEGF(R): vascular endothelial growth factor (receptor)
